# Managing Fever in Children: A National Survey of Parents' Knowledge and Practices in France

**DOI:** 10.1371/journal.pone.0083469

**Published:** 2013-12-31

**Authors:** Nathalie Bertille, Elisabeth Fournier-Charrière, Gérard Pons, Martin Chalumeau

**Affiliations:** 1 Inserm U953 Epidemiological Research Unit on Perinatal Health and Women's and Children's Health, Paris, France; 2 Paris-Descartes University, Paris, France; 3 Department of Paediatrics, Hôpital Necker-Enfants Malades, AP-HP, Paris, France; 4 Pain unit, Hôpital Bicêtre, AP-HP, Bicêtre, France; 5 Clinical Pharmacology, Groupe hospitalier Cochin-Broca-Hôtel Dieu, AP-HP, Paris, France; 6 Inserm U663 Pediatric epilepsies and brain plasticity, Paris, Bicêtre, France; Fondazione IRCCS Ca' Granda Ospedale Maggiore Policlinico, Università degli Studi di Milano, Italy

## Abstract

**Introduction:**

Identifying targets to improve parental practices for managing fever in children is the first step to reducing the overloaded healthcare system related to this common symptom. We aimed to study parents' knowledge and practices and their determinants in managing fever symptoms in children in France as compared with current recommendations.

**Methods:**

We conducted an observational national study between 2007 and 2008 of French general practitioners, primary care pediatricians and pharmacists. These healthcare professionals (HPs) were asked to include 5 consecutive patients from 1 month to 12 years old with fever for up to 48 hr who were accompanied by a family member. Parents completed a questionnaire about their knowledge of fever in children and their attitudes about the current fever episode. We used a multilevel logistic regression model to assess the joint effects of patient- and HP-level variables.

**Results:**

In all, 1,534 HPs (participation rate 13%) included 6,596 children. Parental concordance with current recommendations for temperature measurement methods, the threshold for defining fever, and physical (oral hydration, undressing, room temperature) and drug treatment was 89%, 61%, 15%, and 23%, respectively. Multivariate multi-level analyses revealed a significant HP effect. In general, high concordance with recommendations was associated with high educational level of parents and the HP consulted being a pediatrician.

**Conclusions:**

In France, parents' knowledge and practices related to managing fever symptoms in children frequently differ from recommendations. Targeted health education interventions are needed to effectively manage fever symptoms in children.

## Introduction

Fever is a common symptom in children [1]. It is usually related to a rapidly self-limiting illness and its main effect is discomfort [2]. However, this symptom contributes to overloaded offices of general practitioners (GPs) and office-based pediatricians as well as clinics and pediatric emergency departments [3]. Misconceptions by parents and healthcare professionals (HPs) about rare serious causes such as severe bacterial infections beginning with isolated fever [4] and specific complications of fever (e.g., convulsions) have resulted in a “phobia” about this symptom [2], [3]. To rationalize the symptomatic management of fever in children, several national health agencies and medical societies have disseminated recommendations for HPs and pamphlets for parents with key steps to guide the management of fever symptoms [1], [5]–[7]. These steps include the method for measuring temperature, the threshold for defining fever, indications for starting antipyretic drug treatment, and physical and drug treatments.

Bridging the gap between recommendations and practices (by HPs and parents) could improve the quality of care of children. The first step to bridging this gap is to understand the causes of the gap by measuring the knowledge and practices of parents and HPs related to fever in children. Available studies show that a “fever phobia” has persisted for 40 years since its first description [2], [8], [9], and that parents' knowledge and practices are in poor agreement with some recommendations [2], [3], [10]–[19]. However, these studies do not provide evidence of the current state of knowledge and practices of parents because they pre-date the publication and diffusion of new recommendations [1], [5]–[7], which may have had some impact [3], [9], [10], [12], [14]–[17], [19], [20], or were potentially biased by single-center [9], [11], [14], [21], [22] or hospital-based recruitment [9], [11], [14]–[16], [18], [22]–[24] or a retrospective design [8]–[10], [22]. As well, no studies have considered a highly probable physician or center effect in statistical analyses.

We conducted a national, prospective, cross-sectional study of parents' knowledge and practices related to fever symptoms in their children in France as compared with current recommendations.

## Methods

### Study population

The observational, national study was performed over 8 months from November 2007 to June 2008. We contacted 4,163 GPs with pediatric patients (detected by pediatric vaccine prescriptions) from the national commercial panel of HPs, ICOMED-CEGEDIM [25]; a random sample of French primary care pediatricians; and 4,946 pharmacists (working in the same geographical area as a responding physician). Responding physicians and pharmacists were asked to include 5 consecutive patients from 1 month to 12 years old with fever for up to 48 hr who were accompanied by a family member. In France, pharmacists have an important role in the validation and delivery of prescriptions written by physicians (including over-the-counter drugs), and in counseling patients on drug use and many health topics such as the management of fever in children. After informed consent, parents who agreed to participate completed a written questionnaire asking about their knowledge of fever in children and their practices related to the current fever episode.

### Recommendations

To assess the concordance among parents' knowledge, their attitudes and current recommendations, we reviewed the published recommendations produced in the French language by Western professional groups or societies (in pediatrics, general medicine, emergency care medicine, pharmacology, or pharmacy) or health agencies as well as those produced by the American Academy of Pediatrics [7] and the UK National Institute for Health and Clinical Excellence [1]. In case of disagreement between recommendations (Table 1), we considered concordance with the recommendation(s) produced in the French language and those with the largest dissemination among the population studied (i.e., recommendations of the French drug agency) [6].

**Table 1:** Recommendations for 5 key steps in managing fever in children and parental concordance with recommendations by healthcare professional consulted.

					No. (%) of patients			
5 steps	American Academy of Pediatrics [7]	UK National Institute for Health and Clinical Excellence [1]	French Drug Agency [6]	Canadian Paediatric Society [5]	General practitioners (n = 3,270)	Pediatricians (n = 1,596)	Pharmacists (n = 1,730)	Total (n = 6,596)
**Temperature measurement method**					3270 (100)	1596 (100)	1730 (100)	6596 (100)
Electronic thermometer by rectal route	**X**	**X**	**X**	**X**	1802 (55.1)	1183 (74.1)	1213 (70.1)	4198 (63.6)
Electronic thermometer by oral route	**X**	**X**	**X**	**X**	92 (2.8)	9 (0.6)	30 (1.7)	131 (2.0)
Electronic thermometer by axillary route	**X**	**X**	**X**	**X**	235 (7.2)	114 (7.1)	57 (3.3)	406 (6.2)
Electronic thermometer by aural route	**X**	**X**	**X**	**X**	680 (20.8)	218 (13.7)	321 (18.6)	1219 (18.5)
One of the above methods^a^					2796 (85.5)	1489 (93.3)	1589 (91.8)	5874 (89.0)
Other methods					755 (23.1)	256 (16.0)	424 (24.5)	1435 (11.0)
**Threshold for defining fever**					3265 (99.8)	1593 (99.8)	1730 (100)	6588 (98.9)
<38°C					411 (12.6)	142 (8.9)	204 (11.8)	757 (11.5)
38°C^a^	**X**	**X**	**X**	**X**	1952 (59.8)	1045 (65.6)	1036 (59.9)	4033 (61.2)
>38°C					902 (27.6)	406 (25.5)	490 (28.3)	1798 (27.3)
**Fever threshold for starting antipyretic drug treatment**					3256 (99.6)	1585 (99.3)	1717 (99.2)	6558 (99.4)
<38.5°C					2213 (68.0)	997 (62.9)	1117 (65.1)	4327 (66.0)
38.5°C^a^			**X**		974 (29.9)	550 (34.7)	555 (32.3)	2079 (31.7)
>38.5°C^a^					69 (2.1)	38 (2.4)	45 (2.6)	152 (2.3)
**Physical treatment**					2878 (88.0)	1463 (91.7)	1578 (91.2)	5919 (89.7)
Reduce heating or aerate the room	**X**	**X**	**X**		733 (25.5)	481 (32.8)	396 (25.1)	1610 (27.2)
Undress the child	**X**		**X**	**X**	1731 (60.1)	1096 (74.9)	863 (54.69)	3690 (62.3)
Oral hydration	**X**	**X**	**X**	**X**	2163 (75.1)	1200 (82.2)	1260 (79.8)	4623 (78.1)
All 3 measures^a^	**X**		**X**		375 (13.0)	332 (22.7)	203 (12.9)	910 (15.3)
Other measures					1124 (39.2)	535 (36.6)	764 (48.4)	2427 (41.0)
**Drug treatment**					2972 (90.9)	1527 (95.7)	1533 (88.6)	6032 (91.4)
Monotherapy		**X**	**X**	**X**	2344 (78.9)	1116 (73.1)	980 (63.9)	4400 (73.6)
Monotherapy in 3 to 6 doses per day^a^^, ^ b			**X**	**X**	274 (23.5)	193 (26.8)	116 (17.2)	583 (22.8)
**All recommendations**					6 (0.2)	6 (0.5)	1 (0.1)	13 (0.3)

^a^ Concordance with recommendations of our study.

^b^ For patients with fever for ≥24 hr (n = 2,559).

### Practices

We analyzed 5 key variables (steps) related to knowledge and practices in managing fever symptoms in children: temperature measurement method, threshold for defining fever, fever threshold for starting antipyretic drug treatment (defined only in the recommendations of the French drug agency), and physical and drug treatment [1], [2], [6], [9], [16].

### Determinants

After a literature review and discussions between 3 experts (EFC, GP, MC) in the fields of general pediatrics, pain in children and pediatric pharmacology, we *a priori* considered the following characteristics as potential determinants of concordance with recommendations: (i) at the patient-level, for parents – accompanying parent (mother, father, both or other) and accompanying-parent profession and educational level; and for children – age, gender, and birth rank; and (ii) at the HP-level – profession (GP, pediatrician, pharmacist), practice location (urban, semi-urban, rural), and experience (years of practice).

### Statistical analyses

We described the characteristics of the population (parents and child) and those of the HPs. We calculated concordance for each of the 5 key steps in managing fever by percentages and studied the association of parent-, patient- and HP-level characteristics and concordance with recommendations by univariate analyses. To study the drug administration frequency, we considered only patients having fever for ≥24 hr.

We used a hierarchical regression model that took into account the hierarchical structure of the data (i.e. non-independence of the variables of the 5 patients included by each HP), which allowed for including characteristics of parents and patients at the individual level (level 1) and characteristics of the HP at the HP level (level 2). A different model was constructed for each of the 5 key steps in managing fever symptoms. In each model, we included variables with p≤0.1 on univariate analysis. First, we estimated a random intercept model without any variables (model 1, “empty” model) to obtain the baseline HP-level variance (var^1^) and to test the HP effect. In the second model (model 2), we included patient characteristics, which allowed for investigating the association of patient-level variables and concordance with recommendations and estimating the residual HP-level variation after adjustment for patient-level variables (var^2^). We used the proportional change in variance (PCV = [var^1^-var^2^]/var^1^) to assess the extent to which rate of concordance with recommendations may be explained across differences in distribution of patient-level characteristics. In a third model, we included HP characteristics and estimated the residual HP-level variation after adjustment by patient- and HP-level variables (var^3^) and assessed the extent to which rate of concordance with recommendations may be explained across differences in distribution of HP-level characteristics (PCV = [var^1^-var^3^]/var^1^). Analyses involved use of Stata, v11 (StataCorp, College Station, TX, USA).

### Ethics

Verbal consent from the parents was obtained before inclusion and documented by the completion of the questionnaire. Written consent was not obtained for practical reasons. The study was approved by the Committee of Protection of the Person of Ile-de-France III (N°AT128).

## Results

### Population characteristics

The mean participation rate was 13% (Figure S1): 758 GPs (18%), 374 pediatricians (16%) and 405 pharmacists (8%). We collected 7,619 questionnaires from parents, including 6,596 (87%) that met the inclusion criteria: 4,866 children (74%) from a medical consultation (with 3,270 [50%] seen by GPs and 1,596 [24%] seen by pediatricians), and 1,730 (26%) from a visit to the pharmacy. In all, 93% of the pediatrician recruitments took place in an urban area, as compared with half of the pharmacist and GP recruitments (p<0.001).

For accompanying family members, 95% were one parent, 74% were mothers, and 34% had an educational level above the bachelor's degree, 30% had a bachelor's degree, 35% were employees and 24% were executives. The mean age of patients was 3.7±2.7 years, patients consulting pediatricians were younger (mean 2.5±2.0 years old, p<0.001), and 55% were male. The mean fever level was 38.9±0.6°C. The main other symptoms were pain (64%), flu-like symptoms (49%) and cough (46%).

### Knowledge and practices

For each key step in managing fever symptoms, parents' concordance with recommendations significantly varied by HP (i.e., HP effect, empty model, p<0.001).

For 89% of children, the method parents used to measure temperature (rectal, aural, oral or axillary temperature) complied with recommendations (Table 1). After adjustment for patient and HP characteristics, high educational level of accompanying parent, recruitment by a pediatrician or pharmacist and increased experience of the HP (≥25 years in practice) were associated with high concordance with this recommendation (Table 2, Table S1). Patient-level and HP-level variables explained 12.3% (var^1^ = 0.57, var^2^ = 0.50) and 14.0% (var^3^ = 0.42) of the variance, respectively.

**Table 2:** Summary of actors associated with high concordance with recommendations for the 5 key steps in managing fever symptoms in children (see details including confidence intervals in appendix 2 to 6).

	*5 Steps*				
	Temperature measurement method	Threshold for defining fever	Fever threshold for starting antipyretic drug treatment	Physical treatment	Drug treatment
Factors	*aOR* a	*aOR* a	*aOR* a	*aOR* a	*aOR* a
**Level 1** b					
**Accompanying parent**					
Father vs. mother	NS	0.82*	NS	NS	0.63*
Both parents vs. mother	NS	0.97	NS	NS	0.86
Other vs. mother	NS	0.89	NS	NS	0.70
**Accompanying parent profession**					
Craftsman/storekeeper vs. executive	NS	NS	NS	0.85	0.62*
Employee vs. executive	NS	NS	NS	0.74*	0.80
Salaried worker vs. executive	NS	NS	NS	0.50*	1.01
**Accompanying parent educational level**					
High school graduation vs. postgraduate degree	0.89	NS	0.69*	0.89	NS
Technical school certificate vs. postgraduate degree	0.66*	NS	0.62*	0.70*	NS
Middle school or less vs. postgraduate degree	0.48*	NS	0.53*	0.56*	NS
**No. of children**					
2 vs. 1	NS	NS	NS	0.77*	NS
≥3	NS	NS	NS	0.70	NS
**Child's age**					
1–2.4 years vs. 1–11 months old	NS	NS	1.20	0.90	NS
2.5–4 years vs. 1–11 months old	NS	NS	1.29*	0.79	NS
5 – 12 years vs. 1–11 months old	NS	NS	1.50*	0.70*	NS
**Level 2** c					
**HP** d **profession**					
Pediatrician vs. GP^e^	1.75*	1.19*	1.25*	1.60*	1.05
Pharmacist vs. GP^e^	1.74*	1.00	1.09	1.00	0.69*
**HP** d **experience**					
15–23 vs. ≤14 years in practice	1.16	NS	NS	NS	1.40*
24–54 vs. ≤14 years in practice	1.44*	NS	NS	NS	1.51*

^a^ Adjusted odds ratio (aOR) for patient- or HP-level variables with P<0.1 on univariate analysis.

^b^ At the patient-level, no association was found between any of the 5 steps and the child's gender and birth order.

P≤0.05;

^c^ At the HP-level, no association was found between any of the 5 steps and practice location.

^d^ HP, healthcare professional;

^e^ GP, general practitioner.

For 61% of children, the threshold parents used to define fever complied with recommendations (38°C); for 27%, parents considered an upper threshold, and for 11% a lower one (Table 1). After adjustment for patient and HP characteristics, the accompanying parent being the mother (vs. father) and recruitment by a pediatrician were associated with high concordance with this recommendation (Table 2, Table S2). Patient-level variables explained 10.9% of the variance (var^1^ = 0.19, var^2^ = 0.17) and HP-level variables 6.1% (var^3^ = 0.16).

For 32% of children, parents considered the threshold of 38.5°C recommended by the French drug agency to start a drug treatment to reduce fever symptoms and 66% cited a lower threshold (Table 1). After adjustment for patient and HP characteristics, high educational level of accompanying parent, high age of patient (≥2.5 years) and recruitment by a pediatrician were associated with high concordance with this recommendation (Table 2, Table S3). Patient- and HP-level variables did not explain the HP-level variance (var^1^ = 0.88, var^2^ = 0.92, var^3^ = 0.92).

For 90% of children, parents stated that physical treatment had started before the first contact with the HP and for 95%, this treatment included 1 of the 3 recommended measures (Table 1): oral hydration (78%), undress the child (62%) and lower the heating or aerate the room (27%). Parents for 15% of children used all 3 measures concomitantly as recommended. After adjustment for patient and HP characteristics, accompanying parent profession being an executive (vs. employee or a salaried worker), high educational level, number of children in the family, young age of patient (<5 years) and recruitment by a pediatrician (Table 2, Table S4) were associated with high concordance with this recommendation. Patient-level variables explained 5.9% of the variance (var^1^ = 1.35, var^2^ = 1.27) and HP-level variables 5.2% (var^3^ = 1.20).

For 91% of children, parents had given a drug to lower the fever level of the current episode before the first contact with the HP (Table 1). For 74% of children, in agreement with recommendations, parents who administered an antipyretic gave a single drug. Parents complied more with recommendations (single drug) if they were recruited during a medical consultation than by a pharmacist (77% vs. 64%, p<0.001). Drugs administered by parents were acetaminophen (85%), ibuprofen (13%) and acetylsalicylic acid (1%). Drug treatment had been the objective of a previous prescription for the same child for 74% of children, and drugs were “self-selected” by the parent in the family pharmacy for 28%. For 24% of children who received acetaminophen and 14% of children who received ibuprofen, parents complied with the recommendations (4–6 and 3–4 times a day, respectively). After adjustment for patient and HP characteristics, the accompanying parent being the mother (vs. father), accompanying parent profession an executive (vs. craftsman/storekeeper), recruitment by a GP, and increased experience of the HP (≥15 years in practice) were associated with high concordance with recommendations for drug treatment (Table 2, Table S5). Patient-level variables and HP-level variables explained 2.3% (var^1^ = 0.88, var^2^ = 0.86) and 22.7% (var^3^ = 0.66) of the variance, respectively.

For 13 patients (0.3% of patients with fever for ≥24 hr), parents' knowledge and practices complied with recommendations for all 5 key recommendations for managing fever. Excluding the step fever threshold for starting antipyretic drug treatment (which can be considered specific to the French drug agency's recommendations), 1.2% of parents complied with all recommendations.

## Discussion

In our study, performed in France, parents' knowledge and practices related to managing fever symptoms in their children frequently differ from recommendations. Concordance with recommendations was high for temperature measurement method, moderate for considering the threshold for defining fever, and low for physical and drug treatment. In general, high concordance was associated with high educational level of parents and the HP consulted being a pediatrician. These findings suggest some targets for improving parents' practices for managing fever in their children to avoid unnecessary overload of the healthcare system.

This national non-hospital–based prospective study collected data on nearly 6,600 febrile pediatric patients for increased precision of estimates and power of comparisons between groups. For the first time, a multilevel model was used to take into account the hierarchical structure of the data and the possible association of patient outcomes by HP consulted for fever. Using a hierarchical logistic regression model, we examined the joint effects of patient- and HP-level variables. We also evaluated the extent to which patient- and/or HP-level variables explained the variation in concordance with recommendations by an HP. A conventional regression model could have led to false inferences and would not have allowed for such explorations.

Parents' practice concordance with recommendations varied significantly among the 5 key steps for managing fever symptoms in children. The concordance was adequate (61%) for threshold for defining fever. Interestingly, for 11% of children, parents defined fever by a threshold lower than that recommended (38°C) and for 66%, parents considered a fever threshold for starting antipyretic drug treatment lower than that recommended (38.5°C) by the French drug agency. For 85% of children, parents did not concomitantly carry out the physical treatments recommended (undress the child, oral hydration and lower the heating or aerate the room), and for 77%, parents used a drug administration frequency lower than that recommended (≥3 times a day). These results could explain the prevalent idea that management of fever symptoms is ineffective and could explain the overuse of emergency care providers (consultation without appointment, hospital pediatric emergencies).

Our study confirms an evolution of knowledge and practices of parents. We observed a decrease in the use of baths (23% vs. 33–75% in the literature) [17]–[19], the combination of pharmacological treatments (26% vs. 50–75%) [10], [19], and use of aspirin (1% vs. 4–30%) [10], [19]. However, we observed no improvement in concordance for the threshold for defining fever (61% vs. 40–68%) and adequate method to measure temperature (89% vs. 80–95%) [8]–[11], [17]–[20]. Therefore, it seems that parents' knowledge and practices can be changed, but certain practices may be more difficult to change than others. Our results can be compared with those from a recent survey in another Latin country, Italy [26], where Chiappini et al. found a comparable frequency of use of aspirin (0.5%) and combination of pharmacological treatment (21%) but a lower rate of use of physical treatments (78%) that are not recommended in recent Italian national recommendations [27], [28].

We found a HP effect for all 5 key steps in managing fever symptoms. Among the determinants studied, 1 patient-level variable and 1 HP-level variable were strongly and independently associated with parents' concordance with recommendations: accompanying parents' educational level and HP type. The gradient identified between parents' educational level and concordance with recommendations had been observed in other child health situations [29]. Parents recruited during consultation with a pediatrician complied more with recommendations than those recruited by GPs and pharmacists, which agrees with previous knowledge [30], [31]. Indeed, HPs are important sources of information for parents [10], [12], [16], [17] and pediatricians are more likely to follow recommendations [30], [31] as compared with GPs in various clinical situations. A patient-level variable (accompanying parent educational level) associated with high concordance with recommendations was consistent with that found in previous studies [8], [12], [13], [18], [20], [21]. Nevertheless, the studied variables weakly explained (26% for temperature measurement methods) or not at all the HP-level variations in parents' concordance with recommendations. Variability in parents' concordance by HPs could be explained in part by patient and/or HP characteristics that were not included in our models: sources of information (HP, family and other), quality of information (type, accessibility, and comprehensibility), appropriation of information and barriers to its implementation.

Our study has some limitations. It was conducted in 2008 and an evolution of knowledge and practices is likely. It was based on only reported practices and not observed ones. Selection bias was inherent in the selection of pharmacists and GPs. We could not compare features of participant (13%) and non-participant (87%) physicians and pharmacists, and the number of parents who declined participation is unknown. We cannot estimate the strength and direction of this potential selection bias. Mothers with postgraduate degrees were underrepresented in our sample: 35% vs. 43% in the national perinatal survey of 2003 [32]. A correction by direct standardization of the distribution of educational level in the national perinatal survey of 2003 resulted in a mean increase of 1 point in measured concordance rates (data not shown). We did not consider some groups: parents unable to understand the questions and those who do not usually consult for their child's fever.

## Conclusions

Our study suggests that concordance of parents' knowledge and practices with recommendations for managing fever in their children has improved since the last studies on the subject, varied widely by studied key management steps, and was related to some parent and HP characteristics. Possible health education interventions for more effective management of fever in children could target the concomitant use of all non-drug treatments, drug treatment indications, and drug administration frequency when a drug is started. They could target parents with low educational level (by adjusting the readability of messages and selecting adequate communication media) and some HPs (GPs and pharmacists).

## Supporting Information

Figure S1 **Patients included in the analyses and reasons for exclusion.**
(DOC)

Table S1 Factors associated with temperature measurement method in parents' concordance with recommendations for managing fever in children (rectal, aural, oral or axillary).
(DOC)

Table S2 Factors associated with threshold for defining fever in parents' concordance with recommendations for managing fever in children (38°C).
(DOC)

Table S3 Factors associated with fever threshold for starting antipyretic drug treatment in parents' concordance with recommendations for managing fever in children (≥38.5°C).
(DOC)

Table S4 Factors associated with physical treatments in parents' concordance with recommendations for managing fever in children (oral hydration, undress the child and lower the heating or aerate the room).
(DOC)

Table S5 Factors associated with drug treatments in parents' concordance with recommendations for managing fever in children (monotherapy in 3 to 6 doses per day).
(DOC)
