# Supplementary material for: Managing Fever in Children: A National Survey of Parents' Knowledge and Practices in France
Source: PLoS One. 2013 Dec 31;8(12):e83469. doi: 10.1371/journal.pone.0083469 (PMC3877061; doi:10.1371/journal.pone.0083469)
Supplement: Figure S1 — Patients included in the analyses and reasons for exclusion. (DOC) [file pone.0083469.s001.doc]

Figure S1: Patients included in the analyses and reasons for exclusion
