# Supplementary material for: Managing Fever in Children: A National Survey of Parents' Knowledge and Practices in France
Source: PLoS One. 2013 Dec 31;8(12):e83469. doi: 10.1371/journal.pone.0083469 (PMC3877061; doi:10.1371/journal.pone.0083469)
Supplement: Table S1 — Factors associated with temperature measurement method in parents' concordance with recommendations for managing fever in children (rectal, aural, oral or axillary). (DOC) [file pone.0083469.s002.doc]

Table S1: Factors associated with temperature measurement method in parents’ concordance with recommendations for managing fever in children (rectal, aural, oral or axillary).

|  |  |  |  |  | Multivariate multi-level analyses | | | | |
| --- | --- | --- | --- | --- | --- | --- | --- | --- | --- |
|  |  | Univariate analysis | |  | Model 2 | |  | Model 3 | |
| **Factors** | No. of children | **OR** | **95% CI** |  | **aOR** | **95% CI** |  | **aOR** | **95% CI** |
| **Accompanying parent profession** | |  |  |  |  |  |  |  |  |
| Executive | 1579 | 1 |  |  | 1 |  |  | 1 |  |
| Farmer | 201 | 0.59 | 0.38-0.94 |  | 1.06 | 0.61-1.84 |  | 1.03 | 0.60-1.79 |
| Craftsman/storekeeper | 527 | 0.71 | 0.51-0.99 |  | 0.91 | 0.62-1.34 |  | 0.90 | 0.61-1.32 |
| Employee | 2319 | 0.84 | 0.67-1.06 |  | 1.07 | 0.78-1.47 |  | 1.08 | 0.79-1.47 |
| Salaried worker | 712 | 0.43 | 0.32-0.56 |  | 0.73 | 0.49-1.08 |  | 0.76 | 0.52-1.13 |
| Retired person | 148 | 0.54 | 0.33-0.90 |  | 1.01 | 0.55-1.85 |  | 1.03 | 0.56-1.89 |
| Unemployed | 1040 | 0.45 | 0.35-0.58 |  | 0.82 | 0.56-1.18 |  | 0.84 | 0.58-1.22 |
| **Accompanying parent educational level** | | |  |  |  |  |  |  |  |
| Postgraduate degree | 2273 | 1 |  |  | 1 |  |  | 1 |  |
| High school graduation | 1973 | 0.85 | 0.69-1.06 |  | 0.82 | 0.61-1.09 |  | 0.89 | 0.67-1.19 |
| Technical school Certificate | 1151 | 0.56 | 0.44-0.70 |  | 0.62 | 0.44-0.86 |  | 0.66 | 0.47-0.92 |
| Middle school or less | 1153 | 0.37 | 0.30-0.46 |  | 0.43 | 0.31-0.59 |  | 0.48 | 0.34-0.67 |
| **No. of children** |  |  |  |  |  |  |  |  |  |
| 1 | 2051 | 1 |  |  | 1 |  |  | 1 |  |
| 2 | 2929 | 1.11 | 0.92-1.34 |  | 1.7 | 0.90-1.52 |  | 1.17 | 0.90-1.53 |
| 3 and more | 1509 | 0.65 | 0.53-0.79 |  | 0.69 | 0.48-0.99 |  | 0.72 | 0.50-1.03 |
| **Child’s age** |  |  |  |  |  |  |  |  |  |
| 1–11 months old | 1547 | 1 |  |  | 1 |  |  | 1 |  |
| 1–2.4 years old | 1735 | 0.95 | 0.75-1.21 |  | 0.98 | 0.75-1.26 |  | 1.00 | 0.77-1.29 |
| 2.5–4 years old | 1575 | 0.78 | 0.61-0.98 |  | 0.82 | 0.64-1.06 |  | 0.87 | 0.67-1.13 |
| 5–12 years old | 1739 | 0.62 | 0.50-0.78 |  | 0.71 | 0.55-0.91 |  | 0.77 | 0.60-1.00 |
| **Child’s birth order** |  |  |  |  |  |  |  |  |  |
| First-born | 3200 | 1 |  |  | 1 |  |  | 1 |  |
| Second-born | 2435 | 0.99 | 0.84-1.18 |  | 1.05 | 0.81-1.34 |  | 1.05 | 0.81-1.35 |
| Third-born or more | 878 | 0.74 | 0.59-0.93 |  | 1.29 | 0.88-1.90 |  | 1.32 | 0.90-1.94 |
| **HP profession** |  |  |  |  |  |  |  |  |  |
| General practitioner | 3270 | 1 |  |  |  |  |  | 1 |  |
| Pediatrician | 1596 | 2.36 | 1.89-2.94 |  |  |  |  | 1.75 | 1.34-2.27 |
| Pharmacist | 1730 | 1.91 | 1.57-2.33 |  |  |  |  | 1.74 | 1.38-2.18 |
| **HP experience** |  |  |  |  |  |  |  |  |  |
| 0-14 years in practice | 1955 | 1 |  |  |  |  |  | 1 |  |
| 15-23 years in practice | 2257 | 1.14 | 0.94-1.37 |  |  |  |  | 1.16 | 0.93-1.45 |
| 24-54 years in practice | 2222 | 1.39 | 1.14-1.69 |  |  |  |  | 1.44 | 1.14-1.81 |
| **Variance** |  |  |  |  | 0.50 |  |  | 0.42 |  |
| **PCV^§^ (%)** |  |  |  |  | 12.3 |  |  | 26.3 |  |

Note: OR, odds ratio; 95% CI, 95% confidence interval; HP, healthcare professional

^§^ PCV, **proportional change in variance,** calculated on the basis of the physician-level variance for the empty model (model 1): 0.57 (P<0.001).
