# Supplementary material for: Managing Fever in Children: A National Survey of Parents' Knowledge and Practices in France
Source: PLoS One. 2013 Dec 31;8(12):e83469. doi: 10.1371/journal.pone.0083469 (PMC3877061; doi:10.1371/journal.pone.0083469)
Supplement: Table S2 — Factors associated with threshold for defining fever in parents' concordance with recommendations for managing fever in children (38°C). (DOC) [file pone.0083469.s003.doc]

Table S2: Factors associated with threshold for defining fever in parents’ concordance with recommendations for managing fever in children (38°C).

|  |  |  |  |  | Multivariate multi-level analyses | | | | |
| --- | --- | --- | --- | --- | --- | --- | --- | --- | --- |
|  |  | Univariate analysis | |  | Model 2 | |  | Model 3 | |
| **Factors** | No. of children | **OR** | **95% CI** |  | **aOR** | **95% CI** |  | **aOR** | **95% CI** |
| **Accompanying parent** |  |  |  |  |  |  |  |  |  |
| Mother | 4896 | 1 |  |  | 1 |  |  | 1 |  |
| Father | 1111 | 0.81 | 0.71-0.93 |  | 0.83 | 0.72-0.95 |  | 0.82 | 0.71-0.94 |
| Both parents | 283 | 1.08 | 0.84-1.38 |  | 1.01 | 0.77-1.33 |  | 0.97 | 0.74-1.29 |
| Other | 299 | 0.78 | 0.61-0.98 |  | 0.93 | 0.67-1.29 |  | 0.89 | 0.64-1.24 |
| **Accompanying parent profession** | |  |  |  |  |  |  |  |  |
| Executive | 1579 | 1 |  |  | 1 |  |  | 1 |  |
| Farmer | 200 | 0.82 | 0.61-1.11 |  | 0.87 | 0.64-1.20 |  | 0.87 | 0.63-1.20 |
| Craftsman | 527 | 0.83 | 0.68-1.01 |  | 0.85 | 0.69-1.05 |  | 0.85 | 0.69-1.05 |
| Employed | 2316 | 1.01 | 0.89-1.16 |  | 1.01 | 0.88-1.16 |  | 1.00 | 0.87-1.16 |
| Salaried worker | 712 | 0.83 | 0.69-0.99 |  | 0.84 | 0.70-1.01 |  | 0.85 | 0.70-1.03 |
| Retired person | 148 | 0.65 | 0.46-0.91 |  | 0.69 | 0.43-1.09 |  | 0.72 | 0.45-1.14 |
| Unemployed | 1038 | 0.93 | 0.79-1.10 |  | 0.91 | 0.76-1.07 |  | 0.90 | 0.75-1.07 |
| **Child’s age** |  |  |  |  |  |  |  |  |  |
| 1–11 months old | 1545 | 1 |  |  | 1 |  |  | 1 |  |
| 1–2.5 years old | 1734 | 1.00 | 0.87-1.15 |  | 1.00 | 0.86-1.17 |  | 1.00 | 0.86-1.17 |
| 2.5–5 years old | 1574 | 0.88 | 0.76-1.02 |  | 0.89 | 0.77-1.04 |  | 0.90 | 0.77-1.06 |
| 5–12 years old | 1736 | 0.82 | 0.71-0.94 |  | 0.84 | 0.73-0.98 |  | 0.87 | 0.75-1.02 |
| **HP profession** |  |  |  |  |  |  |  |  |  |
| General practitioner | 3265 | 1 |  |  |  |  |  | 1 |  |
| Pediatrician | 1594 | 1.28 | 1.13-1.45 |  |  |  |  | 1.19 | 1.03-1.39 |
| Pharmacist | 1730 | 1.00 | 0.89-1.13 |  |  |  |  | 1.00 | 0.87-1.14 |
| **HP experience** |  |  |  |  |  |  |  |  |  |
| 0-14 years in practice | 1955 | 1 |  |  |  |  |  | 1 |  |
| 15-23 years in practice | 2256 | 1.08 | 0.96-1.22 |  |  |  |  | 1.06 | 0.92-1.22 |
| 24-54 years in practice | 2216 | 1.17 | 1.03-1.33 |  |  |  |  | 1.13 | 0.98-1.30 |
| **Variance** |  |  |  |  | 0.17 |  |  | 0.16 |  |
| **PCV^§^ (%)** |  |  |  |  | 10.9 |  |  | 17.0 |  |

Note: OR, odds ratio; 95% CI, 95% confidence interval; HP, healthcare professional

^§^ PCV, **proportional change in variance,** calculated on the basis of the physician-level variance for the empty model (model 1): 0.19 (P<0.001).
