# Supplementary material for: Managing Fever in Children: A National Survey of Parents' Knowledge and Practices in France
Source: PLoS One. 2013 Dec 31;8(12):e83469. doi: 10.1371/journal.pone.0083469 (PMC3877061; doi:10.1371/journal.pone.0083469)
Supplement: Table S3 — Factors associated with fever threshold for starting antipyretic drug treatment in parents' concordance with recommendations for managing fever in children (≥38.5°C). (DOC) [file pone.0083469.s004.doc]

Table S3: Factors associated with fever threshold for starting antipyretic drug treatment in parents’ concordance with recommendations for managing fever in children (≥38.5°C).

|  |  |  | |  |  | Multivariate multi-level analyses | | | | |
| --- | --- | --- | --- | --- | --- | --- | --- | --- | --- | --- |
|  |  | Univariate analysis | | |  | Model 2 | |  | Model 3 | |
| **Factors** | No. of children | **OR** | **95% CI** | |  | **aOR** | **95% CI** |  | **aOR** | **95% CI** |
| **Accompanying parent** |  |  |  | |  |  |  |  |  |  |
| Mother | 4872 | 1 |  | |  | 1 |  |  | 1 |  |
| Father | 1105 | 1.15 | 1.00-1.32 | |  | 1.12 | 0.95-1.31 |  | 1.13 | 0.96-1.32 |
| Both parents | 282 | 1.27 | 0.99-1.63 | |  | 1.32 | 0.97-1.80 |  | 1.30 | 0.95-1.76 |
| Other | 299 | 1.11 | 0.87-1.41 | |  | 1.02 | 0.70-1.51 |  | 1.03 | 0.70-1.51 |
| **Accompanying parent profession** | |  |  | |  |  |  |  |  |  |
| Executive | 1571 | 1 |  | |  | 1 |  |  | 1 |  |
| Farmer | 200 | 0.85 | 0.63-1.16 | |  | 1.23 | 0.83-1.83 |  | 1.24 | 0.83-1.84 |
| Craftsman/storekeeper | 523 | 0.93 | 0.76-1.14 | |  | 1.12 | 0.87-1.45 |  | 1.12 | 0.87-1.45 |
| Employee | 2304 | 0.76 | 0.67-0.87 | |  | 1.01 | 0.83-1.23 |  | 1.00 | 0.82-1.22 |
| Salaried worker | 711 | 0.67 | 0.55-0.81 | |  | 1.05 | 0.79-1.40 |  | 1.06 | 0.80-1.41 |
| Retired person | 148 | 0.93 | 0.66-1.32 | |  | 1.30 | 0.75-2.26 |  | 1.30 | 0.75-2.27 |
| Unemployed | 1033 | 0.68 | 0.58-0.81 | |  | 1.03 | 0.80-1.33 |  | 1.03 | 0.80-1.33 |
| **Accompanying parent educational level** | | |  | |  |  |  |  |  |  |
| Postgraduate degree | 2258 | 1 |  | |  | 1 |  |  | 1 |  |
| High School Graduation | 1961 | 0.75 | 0.66-0.85 | |  | 0.67 | 0.56-0.81 |  | 0.69 | 0.57-0.83 |
| Technical School Certificate | 1147 | 0.69 | 0.59-0.80 | |  | 0.60 | 0.48-0.75 |  | 0.62 | 0.49-0.77 |
| Middle school or less | 1147 | 0.62 | 0.53-0.72 | |  | 0.51 | 0.40-0.65 |  | 0.53 | 0.42-0.68 |
| **Child’s gender** |  |  |  | |  |  |  |  |  |  |
| Boy | 3577 | 1 |  | |  |  |  |  | 1 |  |
| Girl | 2937 | 0.91 | 0.82-1.01 | |  | 0.95 | 0.84-1.07 |  | 0.95 | 0.84-1.07 |
| **Child’s age** |  |  |  | |  |  |  |  |  |  |
| 1–11 months old | 1536 | 1 |  | |  |  |  |  | 1 |  |
| 1–2.4 years old | 1724 | 1.12 | 0.97-1.30 | |  | 1.19 | 1.00-1.42 |  | 1.20 | 1.01-1.43 |
| 2.5–4 years old | 1570 | 1.14 | 0.98-1.33 | |  | 1.26 | 1.05-1.51 |  | 1.29 | 1.08-1.55 |
| 5–12 years old | 1728 | 1.28 | 1.11-1.48 | |  | 1.44 | 1.21-1.73 |  | 1.50 | 1.25-1.80 |
| **HP profession** |  |  |  | |  |  |  |  |  |  |
| General practitioner | 3256 | 1 |  | |  |  |  |  | 1 |  |
| Pediatrician | 1585 | 1.25 | 1.10-1.42 | |  |  |  |  | 1.25 | 1.03-1.52 |
| Pharmacist | 1717 | 1.14 | 1.01-1.29 | |  |  |  |  | 1.09 | 0.91-1.31 |
| **Variance** |  |  |  | |  | 0.92 |  |  | 0.92 |  |
| **PCV^§^ (%)** |  |  |  | |  | -4.5 |  |  | -4.5 |  |

Note: OR, odds ratio; 95% CI, 95% confidence interval; HP, healthcare professional

^§^ PCV, **proportional change in variance,** calculated on the basis of the physician-level variance for the empty model (model 1): 0.88 (P<0.001)
