# Supplementary material for: Managing Fever in Children: A National Survey of Parents' Knowledge and Practices in France
Source: PLoS One. 2013 Dec 31;8(12):e83469. doi: 10.1371/journal.pone.0083469 (PMC3877061; doi:10.1371/journal.pone.0083469)
Supplement: Table S4 — Factors associated with physical treatments in parents' concordance with recommendations for managing fever in children (oral hydration, undress the child and lower the heating or aerate the room). (DOC) [file pone.0083469.s005.doc]

Table S4: Factors associated with physical treatments in parents’ concordance with recommendations for managing fever in children (oral hydration, undress the child and lower the heating or aerate the room).

|  |  |  |  | |  | Multivariate multi-level analyses | | | | | | | | |
| --- | --- | --- | --- | --- | --- | --- | --- | --- | --- | --- | --- | --- | --- | --- |
|  |  | Univariate analysis | | |  | Model 2 | | | | |  | | Model 3 | |
| **Factors** | No. of children | **OR** | **95% CI** | |  | **aOR** | | **95% CI** | | |  |  | **aOR** | **95% CI** |
| **Accompanying parent** |  |  | |  |  | |  | |  |  | |  | |  |
| Mother | 4901 | 1 | |  |  | | 1 | |  |  | | 1 | |  |
| Father | 1112 | 0.87 | | 0.71-1.06 |  | | 0.82 | | 0.65-1.04 |  | | 0.83 | | 0.66-1.05 |
| Both parents | 284 | 1.81 | | 1.36-2.42 |  | | 1.57 | | 1.08-2.28 |  | | 1.40 | | 0.96-2.04 |
| Other | 299 | 0.75 | | 0.51-1.09 |  | | 0.91 | | 0.52-1.60 |  | | 0.96 | | 0.55-1.68 |
| **Accompanying parent profession** | |  | |  |  | |  | |  |  | |  | |  |
| Executive | 1579 | 1 | |  |  | | 1 | |  |  | | 1 | |  |
| Farmer | 201 | 0.65 | | 0.43-1.01 |  | | 1.04 | | 0.60-1.82 |  | | 1.15 | | 0.66-2.02 |
| Craftsman/storekeeper | 527 | 0.77 | | 0.58-1.01 |  | | 0.85 | | 0.61-1.20 |  | | 0.85 | | 0.60-1.19 |
| Employee | 2319 | 0.71 | | 0.59-0.84 |  | | 0.75 | | 0.58-0.97 |  | | 0.74 | | 0.57-0.96 |
| Salaried worker | 712 | 0.38 | | 0.28-0.51 |  | | 0.51 | | 0.33-0.77 |  | | 0.50 | | 0.33-0.77 |
| Retired person | 148 | 0.50 | | 0.29-0.86 |  | | 0.74 | | 0.32-1.70 |  | | 0.60 | | 0.25-1.43 |
| Unemployed | 1040 | 0.54 | | 0.43-0.69 |  | | 0.73 | | 0.51-1.03 |  | | 0.72 | | 0.50-1.02 |
| **Accompanying parent educational level** | | | |  |  | |  | |  |  | |  | |  |
| Postgraduate degree | 2273 | 1 | |  |  | | 1 | |  |  | | 1 | |  |
| High school graduation | 1973 | 0.72 | | 0.61-0.86 |  | | 0.82 | | 0.65-1.05 |  | | 0.89 | | 0.69-1.13 |
| Technical school Certificate | 1151 | 0.51 | | 0.41-0.63 |  | | 0.65 | | 0.47-0.88 |  | | 0.70 | | 0.51-0.96 |
| Middle school or less | 1153 | 0.43 | | 0.34-0.54 |  | | 0.51 | | 0.36-0.72 |  | | 0.56 | | 0.39-0.80 |
| **No. of children** |  |  | |  |  | |  | |  |  | |  | |  |
| 1 | 2051 | 1 | |  |  | | 1 | |  |  | | 1 | |  |
| 2 | 2929 | 0.77 | | 0.66-0.91 |  | | 0.76 | | 0.58-0.99 |  | | 0.77 | | 0.59-1.00 |
| ≥ 3 | 1509 | 0.61 | | 0.50-0.75 |  | | 0.69 | | 0.46-1.02 |  | | 0.70 | | 0.47-1.05 |
| **Child’s age** |  |  | |  |  | |  | |  |  | |  | |  |
| 1–11 months | 1547 | 1 | |  |  | | 1 | |  |  | | 1 | |  |
| 1–2.5 years old | 1735 | 0.96 | | 0.80-1.16 |  | | 0.91 | | 0.72-1.14 |  | | 0.90 | | 0.72-1.13 |
| 2.5–5 years old | 1575 | 0.71 | | 0.58-0.87 |  | | 0.74 | | 0.58-0.94 |  | | 0.79 | | 0.62-1.01 |
| 5–12 years old | 1739 | 0.59 | | 0.48-0.72 |  | | 0.64 | | 0.50-0.83 |  | | 0.70 | | 0.54-0.91 |
| **Child’s birth order** |  |  | |  |  | |  | |  |  | |  | |  |
| First-born | 3200 | 1 | |  |  | | 1 | |  |  | | 1 | |  |
| Second-born | 2435 | 0.88 | | 0.76-1.03 |  | | 1.15 | | 0.89-1.48 |  | | 1.16 | | 0.90-1.49 |
| Third-born or more | 878 | 0.68 | | 0.54-0.86 |  | | 1.02 | | 0.66-1.59 |  | | 1.02 | | 0.66-1.59 |
| **HP profession** |  |  | |  |  | |  | |  |  | |  | |  |
| General practitioner | 3270 | 1 | |  |  | |  | |  |  | | 1 | |  |
| Pediatrician | 1596 | 2.03 | | 1.72-2.39 |  | |  | |  |  | | 1.60 | | 1.22-2.09 |
| Pharmacist | 1730 | 1.03 | | 0.86-1.23 |  | |  | |  |  | | 0.94 | | 0.73-1.21 |
| **HP practice location** | |  | |  |  | |  | |  |  | |  | |  |
| Rural | 957 | 1 | |  |  | |  | |  |  | | 1 | |  |
| Largely rural | 1665 | 1.10 | | 0.85-1.42 |  | |  | |  |  | | 1.08 | | 0.77-1.53 |
| Urban | 3885 | 1.50 | | 1.20-1.88 |  | |  | |  |  | | 1.17 | | 0.84-1.62 |
| **Variance** |  |  | |  |  | | 1.27 | |  |  | | 1.20 | |  |
| **PCV^§^ (%)** |  |  | |  |  | | 5.9 | |  |  | | 11.1 | |  |

Note: OR, odds ratio; 95% CI, 95% confidence interval; HP, healthcare professional

^§^ PCV, **proportional change in variance,** calculated on the basis of the physician-level variance for the empty model (model 1): 1.35 (P<0.001).
