# Supplementary material for: Managing Fever in Children: A National Survey of Parents' Knowledge and Practices in France
Source: PLoS One. 2013 Dec 31;8(12):e83469. doi: 10.1371/journal.pone.0083469 (PMC3877061; doi:10.1371/journal.pone.0083469)
Supplement: Table S5 — Factors associated with drug treatments in parents' concordance with recommendations for managing fever in children (monotherapy in 3 to 6 doses per day). (DOC) [file pone.0083469.s006.doc]

Table S5: Factors associated with drug treatments in parents’ concordance with recommendations for managing fever in children (monotherapy in 3 to 6 doses per day).

|  | No. of children |  |  |  | Multivariate multi-level analyses | | | | |
| --- | --- | --- | --- | --- | --- | --- | --- | --- | --- |
|  |  | Univariate analysis | |  | Model 2 | |  | Model 3 | |
| **Factors** |  | **OR** | **95% CI** |  | **aOR** | **95% CI** |  | **aOR** | **95% CI** |
| **Accompanying parent** |  |  |  |  |  |  |  |  |  |
| Mother | 1965 | 1 |  |  | 1 |  |  | 1 |  |
| Father | 401 | 0.68 | 0.52-0.90 |  | 0.66 | 0.48-0.90 |  | 0.63 | 0.46-0.86 |
| Both parents | 99 | 1.07 | 0.67-1.71 |  | 0.96 | 0.54-1.71 |  | 0.86 | 0.49-1.52 |
| Other | 94 | 0.70 | 0.41-1.20 |  | 0.86 | 0.41-1.82 |  | 0.70 | 0.32-1.51 |
| **Accompanying parent profession** | |  |  |  |  |  |  |  |  |
| Executive | 698 | 1 |  |  | 1 |  |  | 1 |  |
| Farmer | 65 | 1.04 | 0.58-1.86 |  | 1.12 | 0.57-2.22 |  | 1.12 | 0.57-2.23 |
| Craftsman/storekeeper | 187 | 0.63 | 0.42-0.95 |  | 0.59 | 0.37-0.94 |  | 0.61 | 0.39-0.98 |
| Employee | 953 | 0.80 | 0.63-1.00 |  | 0.76 | 0.59-0.99 |  | 0.80 | 0.61-1.04 |
| Salaried worker | 225 | 1.00 | 0.71-1.41 |  | 1.01 | 0.68-1.50 |  | 1.01 | 0.68-1.51 |
| Retired person | 38 | 0.35 | 0.12-0.99 |  | 0.35 | 0.90-1.35 |  | 0.46 | 0.12-1.76 |
| Unemployed | 364 | 0.87 | 0.64-1.17 |  | 0.78 | 0.55-1.11 |  | 0.76 | 0.53-1.07 |
| **Child’s age** |  |  |  |  |  |  |  |  |  |
| 1–11 months | 588 | 1 |  |  | 1 |  |  | 1 |  |
| 1–2.4 years old | 683 | 0.99 | 0.77-1.28 |  | 0.97 | 0.72-1.32 |  | 0.93 | 0.69-1.26 |
| 2.5–4 years old | 653 | 0.99 | 0.76-1.28 |  | 0.96 | 0.70-1.30 |  | 0.97 | 0.71-1.32 |
| 5–12 years old | 635 | 0.71 | 0.54-0.94 |  | 0.71 | 0.51-0.98 |  | 0.74 | 0.54-1.03 |
| **HP profession** |  |  |  |  |  |  |  |  |  |
| General practitioner | 1165 | 1 |  |  |  |  |  | 1 |  |
| Pediatrician | 721 | 1.19 | 0.96-1.47 |  |  |  |  | 1.05 | 0.79-1.39 |
| Pharmacist | 673 | 0.68 | 0.53-0.86 |  |  |  |  | 0.69 | 0.51-0.92 |
| **HP experience** | |  |  |  |  |  |  |  |  |
| 0-14 years in practice | 731 | 1 |  |  |  |  |  | 1 |  |
| 15-23 years in practice | 868 | 1.43 | 1.12-1.83 |  |  |  |  | 1.40 | 1.04-1.89 |
| 24-54 years in practice | 899 | 1.51 | 1.19-1.93 |  |  |  |  | 1.51 | 1.13-2.03 |
| **Variance** |  |  |  |  | 0.86 |  |  | 0.66 |  |
| **PCV^§^ (%)** |  |  |  |  | 2.3 |  |  | 25.0 |  |

Note: OR, odds ratio; 95% CI, 95% confidence interval; HP, healthcare professional

^§^ PCV, proportional change in variance, calculated on the basis of the physician-level variance for the empty model (model 1): 0.88 (P<0.001).
